# Alteration of prothrombin time in *Plasmodium falciparum* and *Plasmodium vivax* infections with different levels of severity: a systematic review and meta-analysis

**DOI:** 10.1038/s41598-024-60170-y

**Published:** 2024-05-02

**Authors:** Suriyan Sukati, Tirawat Wannatung, Thitinat Duangchan, Kwuntida Uthaisar Kotepui, Frederick Ramirez Masangkay, Ching-Ping Tseng, Manas Kotepui

**Affiliations:** 1 Medical Technology, School of Allied Health Sciences, Walailak University, Tha Sala, Nakhon Si Thammarat, Thailand; 2 Hematology and Transfusion Science Research Center, Walailak University, Tha Sala, Nakhon Si Thammarat, Thailand; 3 Faculty of Medicine, Western University, Huai Krachao, Kanchanaburi, Thailand; 4 Department of Medical Technology, University of Santo Tomas, Manila, Philippines; 5 Department of Medical Biotechnology and Laboratory Science, College of Medicine, Chang Gung University, Taoyuan, Taiwan; 6 Graduate Institute of Biomedical Science, College of Medicine, Chang Gung University, Taoyuan, Taiwan; 7 Department of Laboratory Medicine, Chang Gung Memorial Hospital, Linkou Branch, Taoyuan, Taiwan; 8 Medical Technology Program, Faculty of Science, Nakhon Phanom University, Nakhon Phanom, Thailand

**Keywords:** Malaria, *Plasmodium*, Prothrombin time, PT, Coagulation, Severity, Diagnostic markers, Prognostic markers, Malaria

## Abstract

Malaria infection leads to hematological abnormalities, including deranged prothrombin time (PT). Given the inconsistent findings regarding PT in malaria across different severities and between *Plasmodium falciparum* and *P. vivax*, this study aimed to synthesize available evidence on PT variations in clinical malaria. A systematic literature search was performed in PubMed, Embase, Scopus, Ovid, and Medline from 27 November 2021 to 2 March 2023 to obtain studies documenting PT in malaria. Study quality was evaluated using the Joanna Briggs Institute checklist, with data synthesized through both qualitative and quantitative methods, including meta-regression and subgroup analyses, to explore heterogeneity and publication bias. From 2767 articles, 21 studies were included. Most studies reported prolonged or increased PT in malaria patients compared to controls, a finding substantiated by the meta-analysis (*P* < 0.01, Mean difference: 8.86 s, 95% CI 5.32–12.40 s, *I*^2^: 87.88%, 4 studies). Severe malaria cases also showed significantly higher PT than non-severe ones (*P* = 0.03, Hedges’s g: 1.65, 95% CI 0.20–3.10, *I*^2^: 97.91%, 7 studies). No significant PT difference was observed between *P. falciparum* and *P. vivax* infections (*P* = 0.88, Mean difference: 0.06, 95% CI − 0.691–0.8, *I*^2^: 65.09%, 2 studies). The relationship between PT and malaria-related mortality remains unclear, underscoring the need for further studies. PT is typically prolonged or increased in malaria, particularly in severe cases, with no notable difference between *P. falciparum* and *P. vivax* infections. The inconsistency in PT findings between fatal and non-fatal cases highlights a gap in current understanding, emphasizing the need for future studies to inform therapeutic strategies.

## Introduction

Malaria, caused by *Plasmodium* species infection, is a serious global health challenge. In 2021, over 200 million people were infected, leading to an estimated 619,000 deaths^1^. Infections by *Plasmodium* species are transmitted through the bites of infected female *Anopheles* mosquitoes^2^. The five most common *Plasmodium* species infecting humans are *Plasmodium falciparum* (*P. falciparum*), *Plasmodium malariae* (*P. malariae*), *Plasmodium vivax* (*P. vivax*), *Plasmodium ovale* (*P. ovale*), and *Plasmodium knowlesi* (*P. knowlesi*), with *P. falciparum* responsible for the most fatal infections. After transmission via mosquito bites, the malaria parasite travels to the liver, replicates in the host's liver cells, and then proceeds to infect red blood cells in the circulation^3^. *Plasmodium* infection can lead to a wide range of clinical symptoms, which may include fever, chills, headaches, nausea, vomiting, muscle aches, fatigue, and, in some cases, jaundice due to the destruction of red blood cells. Symptoms can vary from mild, uncomplicated malaria, which involves the aforementioned symptoms without signs of severe organ dysfunction, to severe malaria, characterized by more serious conditions such as impaired consciousness, respiratory distress, multiple convulsions, severe anemia, hemoglobinuria, acute kidney injury, hyperparasitemia, and complications leading to death^4^. Well-documented hematological changes occur during malaria infection, such as anemia, thrombocytopenia, and lymphocytopenia^5,6^. Moreover, severe malaria can result in significant liver damage, respiratory distress, renal failure, multi-organ dysfunction, abnormal bleeding, and cerebral malaria—a fatal neurological complication predominantly associated with *P. falciparum* infection^7^.

Coagulation, a critical process in hemostasis, involves a complex cascade of events that lead to the formation of a blood clot, preventing excessive bleeding when blood vessels are injured^8,9^. Initially, the process begins with the vascular injury, triggering the exposure of tissue factor, a key activator of the coagulation cascade. This exposure leads to the activation of the extrinsic pathway, marked by the conversion of prothrombin to thrombin. Thrombin then plays a central role in converting fibrinogen into fibrin, which forms the structural basis of a clot^10^. Simultaneously, the intrinsic pathway, initiated by contact activation factors within the blood (Factors XII, XI, VIII, IX), converges with the extrinsic pathway to amplify thrombin production. The common pathway, involving both extrinsic and intrinsic pathways, culminates in the stabilization of the fibrin clot alongside activated platelets, effectively sealing the site of vascular injury^8–10^.

Coagulation abnormalities are common laboratory findings in malaria infection, observable across the spectrum from uncomplicated to severe malaria^11,12^. Previous studies have reported that clinically apparent bleeding and disseminated intravascular coagulation (DIC), a severe coagulopathy, is associated with severe malaria infection and is more common in cases of cerebral malaria^13,14^. It has been reported that approximately 5–10% of severe malaria cases develop DIC, which is associated with high mortality^15^. The pathogenesis of *Plasmodium* species, specifically, *P. falciparum* is known to cause severe endothelial dysfunction in cases of both uncomplicated and fatal malaria due to its unique ability to sequester in the microvasculature and induce inflammatory responses^11^. Various procoagulants present during malaria infection, including tissue factor (TF) released from damaged vascular endothelial cells, the induction of TF expression in endothelial cells, exposed phosphatidylserine on infected red blood cells, lysis of activated platelets, macrophage migration inhibitory factor (MIF), and interleukin-6 cytokine (IL-6), are believed to activate the coagulation system^16–18^.

Routine laboratory coagulation tests, such as prothrombin time (PT) and activated partial thromboplastin time (APTT), are commonly used to investigate blood coagulation changes. PT reflects the activity of the extrinsic (factor VII) and common (factors V, X, II, and I) coagulation pathways, while APTT monitors the competency of the intrinsic (Factors XII, XI, VIII, IX) and common pathways^19^. Prior research indicates that the extent of coagulation derangement often corresponds with the severity and activity of the disease process^20^. Some studies indicate that patients with high parasitemia often experience alterations in coagulation tests, as high parasitemia is known to enhance fibrin formation and activate plasminogen, thus disturbing the coagulation system^21–23^. Notably, prolonged PT has been observed in *P. falciparum* infections characterized by high parasitemia^22^, suggesting that PT could potentially serve as a predictive marker for the clinical progression of *Plasmodium* infection. Nevertheless, the results of PT in malaria remain inconsistent across different studies. Therefore, this systematic review and meta-analysis aims to investigate differences in PT between malaria patients and uninfected controls, between varying degrees of disease severity, between different *Plasmodium* species, and between fatalities and survivors. Such a data collection would help in monitoring and predicting disease progression and could aid in preventing life-threatening complications of malaria infection through early detection and proper management of the hypercoagulable state.

## Methods

### Protocol and registration

The protocol of systematic review was registered at PROSPERO with a registration number CRD42022346003. The systematic review followed Preferred Reporting Items for Systematic Reviews and Meta-analyses (PRISMA) guidelines^24^.

### Data sources and searches

A comprehensive search was conducted in databases such as PubMed, Embase, Scopus, Ovid, and Medline from their inception up to March 2, 2023 (the final date of searches), to identify studies reporting on PT in malaria cases. To construct the search strategy, the search terms were combined with Boolean operators (AND, OR): (coagulation OR "blood clotting" OR "prothrombin time" OR prothrombin OR "russell viper venom time" OR "russells viper venom time" OR "thrombotest" OR "quick test"). Language restrictions were applied to include only English-language articles, without constraints on the year of publication. The details of the searches in all databases are listed in Table S1.

### Definitions

Severe *P. falciparum* malaria is characterized by the presence of *P. falciparum* asexual parasitaemia alongside one or more complications as specified by the WHO criteria for severe malaria such as impaired consciousness, prostration, severe malarial anemia, jaundice, acidosis, renal impairment, significant bleeding, shock, multiple convulsions, hypoglycemia, and hyperparasitemia^25^. Severe *P. vivax* malaria follows the same definition as severe *P. falciparum* malaria, except that it doesn't require any specified parasite density thresholds. Non-severe malaria, on the other hand, is identified by the detection of asexual *Plasmodium* parasitemia, yet with the absence of any complications stipulated by the WHO's criteria for severe malaria.

### Outcomes

The outcomes of the systematic review were the difference in PT between the following groups of participants: (i) malaria cases and uninfected controls, (ii) severe and non-severe malaria cases, (iii) different *Plasmodium* species, and (iv) deaths and survivors.

### Eligibility criteria

The PICo (P: population, I: Outcome of interest, Co: context) approach was used to include eligible studies. (i) P: patients with malaria in all clinical severity (asymptomatic, uncomplicated, severe, or fatal malaria). (ii) I: prothrombin time in qualitative (prolonged or normal) and quantitative using mean ± standard deviation (SD) for normally distributed data or median and interquartile range (IQR) for data not normally distributed. Co: worldwide or global. The inclusion criteria are: (i) original studies that investigated prothrombin time in patients with malaria; (ii) study designs that could be cross-sectional, cohort, or case–control studies; (iii) PT assessments conducted upon admission, before treatment. The exclusion criteria were: case reports/case series, clinical trials without baseline PT measurements, letters, communications, conference abstracts, book series, in vitro studies, articles not in English, and reviews/systematic reviews.

### Study selection and data extraction

Study selection was performed by two review authors (MK and SS) independently using EndNote 20 for reference management. First, duplicate studies were excluded, and then titles and abstracts of the remaining studies were screened. Second, studies with irrelevant titles and abstracts were removed. Third, potentially relevant studies were examined for full-text, and ineligible studies were removed with specific reasons. Fourth, studies that met the eligibility criteria were included in the systematic review. After the studies were selected based on the eligibility criteria, the following data were extracted from each study using a pilot-tested, standard datasheet: name of the first author, publication year, study design, study location (year of conduction), characteristics of participants enrolled, *Plasmodium* spp., age range, PT in malaria and other groups, method for malaria detection, and method for PT. Data from each study were extracted by two authors (SS and MK). Any disagreements regarding study selection and data extraction between the two authors were resolved through discussion to reach a consensus.

### Quality assessment

Two review authors (SS and TD) independently assessed the quality of included studies using the Joanna Briggs Institute (JBI) critical appraisal checklist for observational studies^26^. The checklist contains a set of questions that determine the internal and external validity of a study. These questions cover various aspects of study design, conduct, and reporting, including: sampling of study participants, sample size, study subjects and the setting, data analysis, methods used for the identification of the condition, a standard, reliable way for measurement of the condition, statistical analysis, confounding factors, identification of subpopulations, and measurement of outcome. Each item is scored as "yes," "no," "unclear," or "not applicable". The total score defines the overall quality of the study in which low, moderate, and the high quality if the total score were ≤ 50%, 51–74%, and ≥ 75%, respectively.

### Data synthesis

Data synthesis was performed using qualitative and quantitative approaches. The qualitative involved the narrative description of the results of an individual study; meanwhile, the quantitative synthesis involved the pooling of results from several studies that reported the same outcome data. In the present study, the pooled mean difference (MD) or standardized mean difference (Hedge’s g) was used as the pooled effect estimates of PT between groups of participants including (i) malaria cases and uninfected controls, (ii) severe and non-severe malaria cases, (iii) and different *Plasmodium* species. The heterogeneity of the effect estimates were determined using the I^2^ for inconsistency in which I^2^ values between 25–50%, 51–75%, and > 75% indicated low, moderate, and significant heterogeneity, respectively^27^. In the meta-analysis comparing PT between groups of participants, the meta-regression and subgroup analyses were conducted to identify the source of the heterogeneity. These analyses were stratified based on several factors such as publication year, study design, country, continent, *Plasmodium* species, age groups, methods for malaria diagnosis, and methods for PT assessment. For any comparison involving more than ten studies, the potential publication bias and the influence of small-study effects were assessed using the funnel plots, contour-enhanced funnel plots, and Egger’s regression test^28^. All the computations were performed using Stata software version 17.0 (StataCorp LLC, College Station, TX).

## Results

### Search results

**Figure 1:**
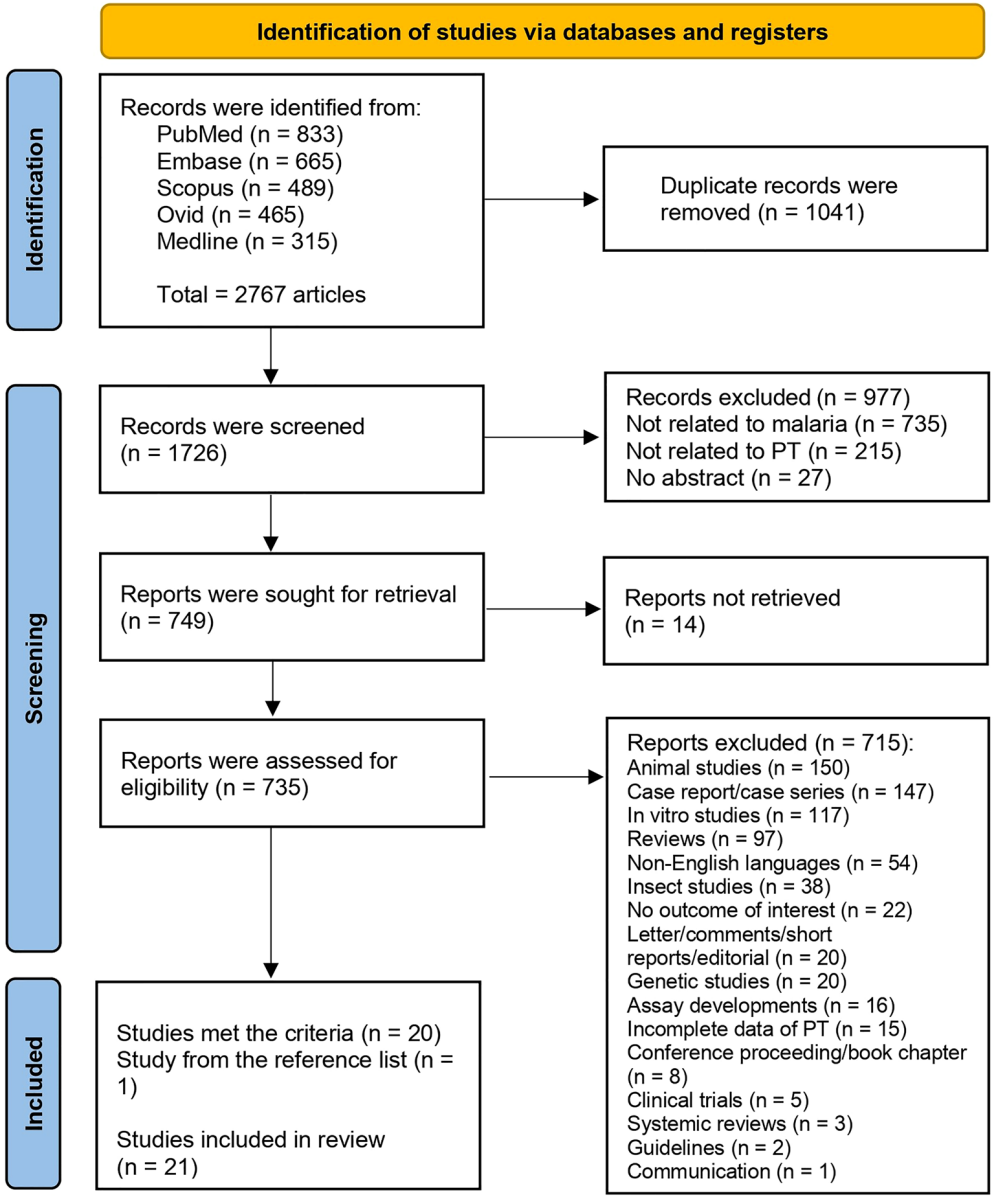
Study flow diagram showing the study selection processes.

From the identified 2767 articles across databases including PubMed, Embase, Scopus, Ovid, and Medline, 1041 duplicates were removed. This resulted in 1726 unique records, which were screened for relevance. Subsequently, 977 records not relevant to malaria, PT, or lacking abstracts were eliminated. Of the 749 reports marked for retrieval, 14 were inaccessible, leaving 735 for eligibility assessment. After rigorous evaluation, 715 were further excluded due to a variety of reasons including irrelevance to the study's focus, being animal/in vitro studies, reviews, non-English language studies, among others. Ultimately, 20 studies that met the selection criteria were identified. An additional study, identified from a reference list, was included, totaling 21 studies for review^29–49^ (Fig. 1).

### Characteristics of the included studies

Table 1 summarize the characteristics of the 21 studies included in the review. Out of 21 studies analyzed, the majority (52.38%) were published between 2010 and 2019, with the common study design being retrospective (33.33%). Most research was conducted in Asia (61.90%), specifically India (38.1%). The *Plasmodium* species most studied was *P. falciparum* (42.86%). Study participants were mainly adults (33.33%) or unspecified (33.33%). Microscopy (52.38%) was the dominant method for malaria detection, while the majority of studies (66.67%) did not specify their PT measurement method (Table 1). Details of all included studies were demonstrated in Table S2.

**Table 1:** Summary characteristics of studies.

Characteristics	N. (21 studies)	%
Publication year		
2020–2023	4	19.25
2010–2019	11	52.38
2000–2009	3	14.29
Before 2000	3	14.29
Study designs		
Retrospective study	7	33.33
Prospective study	6	28.7
Cross-sectional studies	4	19.05
Cohort study	3	14.29
Case–control studies	1	4.67
Study areas		
Asia	13	61.90
India	8	38.10
Pakistan	3	14.29
Israel	1	4.76
Singapore	1	4.76
Africa	6	28.57
Malawi	2	9.52
Congo	1	4.76
Gabon	1	4.76
Sudan	1	4.76
Nigeria	1	4.76
Europe	2	9.52
England	1	4.76
Germany	1	4.76
*Plasmodium* species		
*P. falciparum*	9	42.86
*P. falciparum, P. vivax*	3	14.29
*P. falciparum, P. vivax*, mixed infections	3	14.29
*P. vivax*	2	9.52
*P. falciparum*, non-*P. falciparum*	1	4.76
*P. vivax*, mixed infections	1	4.76
Not specified	2	9.52
Participants		
Adults	7	33.33
Children	4	19.25
All age groups	3	14.29
Not specified	7	33.33
Methods for malaria detection		
Microscopy	11	52.38
Microscopy/RDT	7	33.33
Microscopy/RDT/PCR	1	4.76
Not specified	2	9.52
Method for PT measurement		
The manual clotting method	4	19.05
The optical clotting method by automation	3	14.29
Not specified	14	66.67

### Quality of the included studies

For case–control studies, two studies^29,33^ showed issues in group comparability regarding the presence or absence of disease (Table S3). Despite meeting the criteria for appropriate matching of cases and controls, the same identification criteria, and standard exposure measurement, both studies lacked the identification of and strategies for dealing with confounding factors. For cross-sectional studies, one study was included in the appraisal despite not clearly defining inclusion criteria, failing to identify confounding factors, and lacking appropriate statistical analysis^31^. One study, on the other hand, met all appraisal criteria^35^. Two studies^43,48^, while failing to fully address confounding factors, were also included due to their adherence to the remaining criteria.

For cohort studies, a significant number of studies all demonstrated a similar pattern^30,32,34,36–38,40–42,46,47,49^. They met most of the appraisal criteria but failed to identify or state strategies to handle confounding factors and did not employ strategies to address incomplete follow-up. Despite these shortcomings, they were all included in the appraisal. One study managed to identify and address confounding factors but did not manage to address incomplete follow-up^39^. One study did not adequately address confounding factors or incomplete follow-up and failed to use appropriate statistical analysis, yet was included^44^. Lastly, one study^45^ faced a unique issue with unclear exposure measurement but was included despite this and the usual issues with confounding factors and incomplete follow-up.

### Difference in PT between malaria patients and uninfected controls

**Figure 2:**
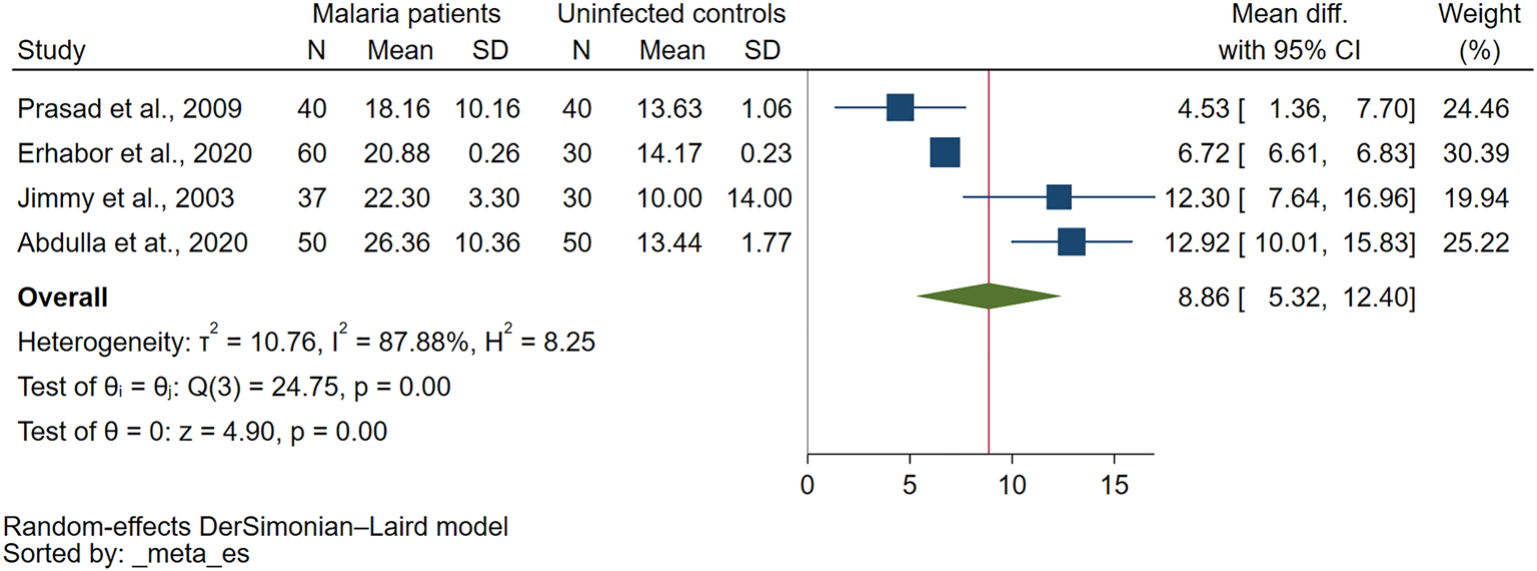
The meta-analysis using the data from four studies showed significantly higher PT (unit in second) in malaria patients compared to uninfected controls. Abbreviations: N, number of participants; CI, confidence interval; SD, standard deviation.

Comparison of PT between malaria patients and uninfected controls were reported in eight studies^29,31,33,34,36,45–47^. Six studies demonstrated that PT was significantly higher in malaria patients than uninfected controls^29,33,34,36,46,47^ while two studies reported that PT was prolonged in uncomplicated malaria (11/47 cases) as compared to normal uninfected controls^31^. PT was prolonged only in cerebral malaria but normal in non-cerebral severe malaria, uncomplicated malaria, non-malaria, and healthy controls^45^. The meta-analysis using the data from four studies that reported quantitative data of PT^29,33,36,47^ showed a significantly higher PT in malaria patients as compared to uninfected controls (*P* < 0.01, Mean difference: 8.859 s, 95% CI 5.315–12.403 s, *I*^2^: 87.88%, 4 studies Fig. 2).

### Difference in PT between severe and non-severe malaria

**Figure 3:**
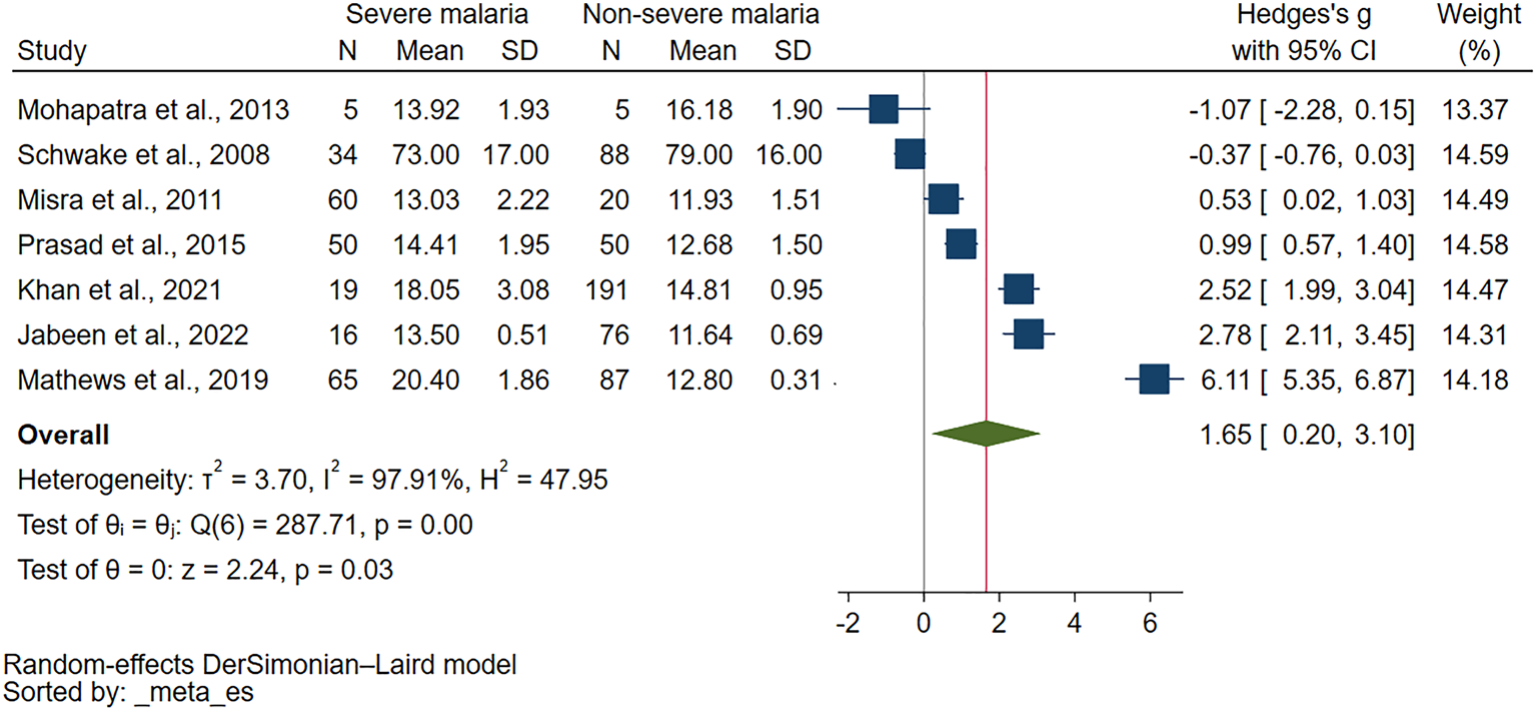
The meta-analysis using the data from seven studies showed significantly higher PT (unit in second) in severe malaria than non-severe malaria. Abbreviations: N, number of participants; CI, confidence interval; SD, standard deviation.

Comparison of PT in severe and non-severe malaria were reported in 11 studies^31,35,37,40–45,48,49^. Out of 11 studies examining PT in severe and non-severe malaria, varied results were reported. Several studies^35,37,40,42,48^ found PT to be higher in severe malaria than non-severe cases. One study found no significant difference in PT between severe and non-severe malaria^49^, while another reported normal PT levels in both conditions^41^. Interestingly, one study reported normal PT in severe malaria but prolonged PT in non-severe malaria^31^. Another study found prolonged PT in cerebral malaria but normal in non-cerebral severe malaria and non-severe malaria^45^, whereas one found prolonged PT in cerebral severe malaria but normal in non-severe malaria^43^. Finally, one study reported prolonged PT in both severe and non-severe malaria^44^. The meta-analysis using the data from seven studies that reported quantitative data of PT^35,37,40,42,44,48,49^ showed a significantly higher PT in severe malaria than non-severe malaria (*P* = 0.03, Hedges's g: 1.65, 95% CI 0.20–3.10, *I*^2^: 97.91%, 7 studies Fig. 3).

The meta-regression analysis was further performed to identify the source of heterogeneity of the effect estimate. The results showed that publication years, study design, country, continent, age group, *Plasmodium* species, diagnostic method for malaria, or method for PT measurement did not affect the pooled effect estimate (Table S4). Although these covariates did not affect the pooled effect estimate, the R^2^ values indicated that the covariate, diagnostic method for malaria, explains 11.15% of the variation in PT differences between severe and non-severe malaria cases, indicating some level of explanatory power, though still relatively low. The subgroup analysis has been further performed to identify the source of heterogeneity of the effect estimate (Table 2). The subgroup analysis based on publication years showed no difference in PT for studies published from 2010 to 2019 (*P* = 0.18), while those from 2000 to 2009 reported a significant increase in PT (*P* < 0.01). The subgroup analysis based on study design showed that cross-sectional study subgroup yielded a significant increase in PT (*P* = 0.04), while prospective studies subgroup showed no difference in PT (*P* = 0.12). The subgroup analysis based on continent found a significant increase in PT for severe malaria cases in Asia (*P* = 0.01). The subgroup analysis based on age showed a trend toward higher PT in adults with severe malaria, but no conclusive results for unspecified age or all age groups due to insufficient data. In the context of *Plasmodium* species, studies reporting on *P. falciparum* indicated a non-significant difference in PT (*P* = 0.38). *P. vivax* studies revealed a non-significant difference in PT (*P* = 0.48). Subgroup analysis based on diagnostic methods demonstrated a significant increase in PT for severe malaria when Microscopy/RDT was used (*P* = 0.03), but not when Microscopy alone was used (*P* = 0.65). Lastly, the method of PT measurement subgroup analysis showed a significant increase in PT in severe malaria for studies where the PT measurement method was not specified (*P* = 0.01). The manual and optical clotting methods could not generate *P*-values due to insufficient data.

**Table 2:** Subgroup analyses of PT between severe and non-severe malaria.

Subgroup analyses	*P* value	Hedges’ g, 95% CI	I^2^ (%)	Number of studies
Publication years				
2020–2023	N/A	− 0.37, − 0.76–0.03	N/A	1
2010–2019	0.18	1.66, 0.75–4.06	98.33	4
2000–2009	< 0.01	2.62, 2.20–3.03	0	2
Study design				
Cohort study	N/A	− 0.37, − 0.76–0.03	N/A	1
Cross-sectional study	0.04	1.86, 0.11–3.62	95.00	2
Prospective study	0.12	2.04, − 0.50–4.59	98.27	4
Continent				
Asia	0.01	2.00, 0.45–3.55	97.50	6
Europe	N/A	− 0.37, − 0.76–0.03	N/A	1
Age group				
Adults	0.09	1.79, − 0.31–3.89	98.66	4
All age groups	N/A	2.52, 1.99–3.04	N/A	1
Not specified age	0.64	0.89, − 2.88–4.66	96.62	2
*Plasmodium* species				
*P. falciparum*	0.38	0.38, − 0.46–1.22	90.99	3
*P. vivax*	0.48	2.54, − 4.49–9.57	98.97	2
*P. falciparum, P. vivax*	N/A	2.78, 2.11–3.45	N/A	1
*P. falciparum, P. vivax,* mixed infections	N/A	2.52, 1.99–3.04	N/A	1
Diagnostic method for malaria				
Microscopy	0.65	0.31, − 1.02–1.64	95.35	2
Microscopy/RDT	0.03	2.20, 0.23–4.17	97.73	5
Methods for PT measurement				
The manual clotting method	N/A	− 1.07, − 2.28–0.15	N/A	1
The optical clotting method by automation	N/A	0.53, 0.02–1.03	N/A	1
Not specified	0.01	2.39, 0.551–4.26	98.47	5

*CI* confidence interval, *N/A* not assessed, *RDT* rapid diagnostic test.

### Difference in PT between different *Plasmodium* species

Comparison of PT in different *Plasmodium* species were reported in six studies^30,32,35,37,39,41^. There appears to be variance in the PT based on the *Plasmodium* species involved in the malaria infection. For instance, one study suggested PT prolongation in both *P. vivax* and *P. falciparum* malaria^32^. Contrarily, another study found PT to be prolonged in *P. falciparum* malaria, but normal in *P. vivax* malaria and mixed infections^39^. In mixed infection cases, PT was found to be significantly higher than in *P. vivax* mono-infection cases^30^. However, another investigation reported normal PT for both *P. falciparum* and *P. vivax* malaria^41^. Two additional studies did not provide qualitative results comparing PT between different *Plasmodium* species, but they provided quantitative data for the meta-analysis ^35,37^. There was no significant difference in PT between patients with *P. falciparum* and those with *P. vivax* mono-infection (*P* = 0.88, Mean difference: 0.06, 95% CI − 0.691–0.8, *I*^*2*^: 65.09%, 2 studies, Supplementary Fig. 1). There was no significant difference in PT between patients with *P. falciparum*/*P. vivax* mixed infections and those with *P. vivax* mono-infection (*P* = 0.35, Mean difference: 4.37 s, 95% CI − 4.77–13.52 s, *I*^2^: 99.21%, 2 studies, Supplementary Fig. 2).

### Difference in PT between fatal malaria and non-fatal malaria

The comparison of PT in fatal and non-fatal malaria cases has been addressed in two studies^31^. One study noted a significantly higher PT in fatal malaria cases compared to surviving cases^38^. Contrarily, another study reported PT as normal in instances of fatal malaria^31^.

### Publication bias

Publication bias was not assessed using funnel plots, contour-enhanced funnel plots, or Egger’s regression test because the meta-analysis included fewer than ten studies.

### Sensitivity analysis

**Figure 4:**
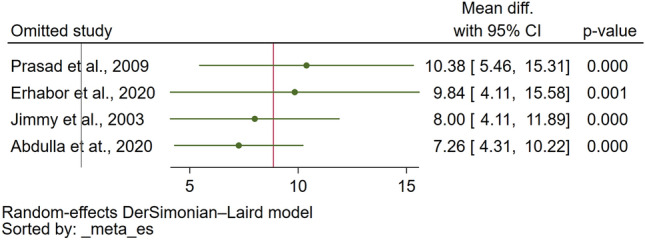
The leave-one-out meta-analysis of PT (unit in second) between malaria patients and uninfected controls showed no study outlier that affect the pooled effect estimate. Abbreviations: CI, confidence interval.

**Figure 5:**
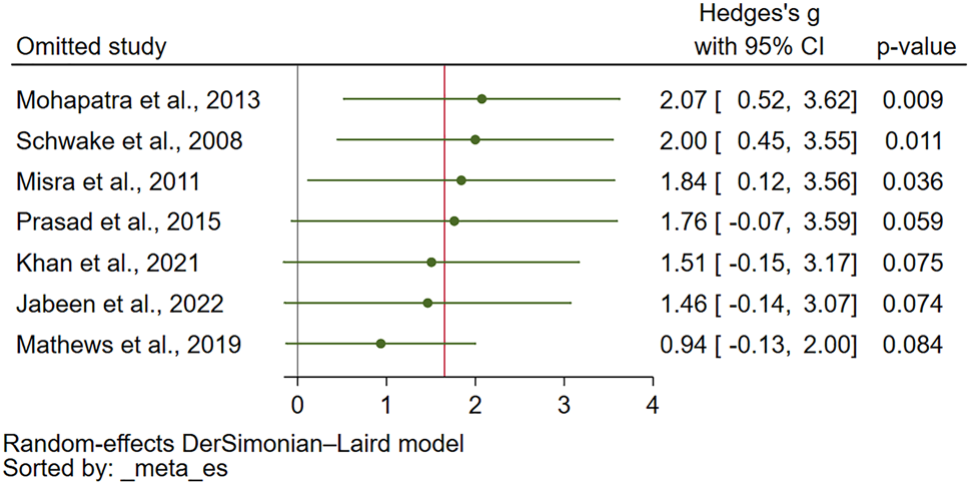
The leave-one-out meta-analysis of PT (unit in second) between severe and non-severe malaria showed study outliers that affect the pooled effect estimate. Abbreviations: CI, confidence interval.

The sensitivity analysis was conducted to assess the robustness of the meta-analysis findings. For assessing PT differences between malaria patients and uninfected controls, the meta-analysis demonstrated stability under both the fixed effect model and when calculating the standard mean difference (SMD) (Supplementary Figs. 3 and 4, respectively). Furthermore, the leave-one-out sensitivity analysis did not identify any single study as an outlier that significantly influenced the overall pooled effect estimate (Fig. 4), affirming the consistency of these results. Similarly, the stability of the meta-analysis comparing PT between severe and non-severe malaria cases was evaluated. When the fixed effect model was applied, the analysis remained stable (Supplementary Fig. 5). However, the leave-one-out sensitivity analysis revealed the presence of study outliers that had a notable impact on the pooled effect estimate (Fig. 5). This highlights certain studies' influence on the overall meta-analysis outcome for PT differences between severe and non-severe malaria cases.

## Discussion

Malaria's impact on blood coagulation, evidenced by alterations in prothrombin time (PT), underscores a complex interplay between the disease's pathophysiology and coagulation pathways^11^. The meta-analysis results also supported the finding from individual study in which PT was statistically increased in malaria patients as compared to uninfected controls. Furthermore, the meta-analysis result was stable when the statistical model or the pooled effect estimate were changed, indicating a certain degree of coagulation activation that causes the alteration of PT during *Plasmodium* infections. In uncomplicated malaria, prolonged PT could be observed between 3.77 and 16.7% in different studies^39,50,51^. The presence of a prolonged PT, along with clinical indications such as bleeding manifestations and standard coagulation laboratory tests such as APTT, D-dimers, and fibrinogen degradation products, can suggest the onset of DIC. Consequently, DIC can trigger both microvascular and macrovascular clotting, impairing blood circulation, which can ultimately result in the development of Multiple Organ Dysfunction Syndrome (MODS)^52^.

The systematic review revealed that patients suffering from severe malaria exhibited a prolonged PT in comparison to those with non-severe malaria. This finding was statistically significant, with PT levels being higher in severe malaria patients than in uninfected controls. Such observations align with the existing literature that links severe malaria, especially cases involving *P. falciparum*, to significant disruptions in coagulation pathways^47^. Specifically, *P. falciparum*-infected erythrocytes are known to exhibit elevated levels of tissue factor^16^, a key initiator of the coagulation cascade, which can lead to increased PT. The activation of the coagulation system, coupled with the impairment of anticoagulant mechanisms, contributes to a prothrombotic state^11^, thereby explaining the prolonged PT observed in severe malaria cases. This activation of the coagulation system, along with impaired anticoagulant mechanisms, likely contributes to a prothrombotic state, elucidating the extended PT observed in cases of severe malaria. Numerous studies have documented a trend towards more pronounced PT alterations in patients with severe malaria compared to those presenting with uncomplicated forms of the disease^20,32,43,47,53–55^.

The PT prolongation in severe malaria, observed in the range of 5.74–22.7%^56–59^, could be attributed to several mechanisms. One such mechanism might include the release of tissue factors from damaged vascular endothelial cells or the expression of activated tissue factor on endothelial cells, leading to consumptive coagulopathy due to the activation of the extrinsic coagulation pathway^16,60^. Nevertheless, certain studies showed that the proportion of prolonged PT was similar in both severe and uncomplicated malaria, or there was no significant difference in PT between the two clinical malaria groups^41,44,49,61^. Inconsistency was observed in the meta-analysis results, as alterations in the statistical model or the pooled effect estimate led to instability in the outcomes. However, based on the available evidence, it can be concluded that patients with severe malaria are more likely to exhibit prolonged or increased PT.

The systematic review highlighted the limited evidence differentiating PT between *Plasmodium* species. According to the meta-analysis, there were observed similarities in PT between patients with *P. falciparum* mono-infection and those with *P. vivax* mono-infection, as well as between patients with *P. falciparum*/*P. vivax* mixed infections and those with *P. vivax* mono-infection. These findings suggest that an increase or prolongation in PT can occur in *P. falciparum*, non-*P. falciparum* malaria, and mixed *Plasmodium* infections. Interestingly, prolonged PT was observed in patients suffering from both severe *P. falciparum* and severe *P. vivax* malaria. In a study that involved both *P. vivax* and *P. falciparum*^59^, there was a minimal difference in the proportion of prolonged PT, with severe *P. falciparum* malaria having a slightly higher proportion of prolonged PT than severe *P. vivax* malaria (10.6 vs. 5.74%). The slight variance in PT prolongation rates between these two *Plasmodium* species suggests that underlying mechanisms, such as differences in endothelial activation and microvascular dysfunction^62^, may contribute to this discrepancy. Nonetheless, the specific factors driving these subtle differences remain to be fully elucidated, highlighting an area for future research.

The systematic review of studies involving patients with both fatal and non-fatal malaria demonstrated varied results. The discrepancies in these findings may be attributable to the multifaceted nature of the factors contributing to the risk of death among malaria patients. For instance, those suffering from fatal malaria could potentially experience a higher consumption of coagulation factors than patients with non-fatal malaria^63^. This enhanced consumption of coagulation factors in fatal malaria cases may be linked to a heightened degree of coagulation activation. This heightened activation, in turn, could be driven by systemic endothelial activation, a common occurrence in severe or fatal malaria^64^. This scenario proposes a complex interplay between coagulation factors and the pathophysiology of malaria, suggesting that severe or fatal malaria cases exhibit a hyperactive coagulation state, leading to excessive consumption of coagulation factors, subsequently resulting in a more prolonged PT. However, it is important to underscore that while the correlation between fatal malaria and heightened coagulation activation seems plausible, the evidence is not wholly conclusive. The heterogeneous nature of these findings underscores the need for more in-depth investigations. Future studies should aim to elucidate the precise mechanisms by which malaria influences the coagulation pathway and the subsequent variations in PT, as well as the potential discrepancies in these effects between fatal and non-fatal cases.

The systematic review and meta-analysis of PT in malaria patients have several limitations. First, the significant heterogeneity observed across studies may compromise the validity of the findings. Despite the subgroup analysis and meta-regression performed to identify the source of heterogeneity, the variance remained largely unexplained, which implies that other factors unaccounted for in this study might be contributing to this heterogeneity. Second, the studies used for comparison were not homogenous in terms of patient characteristics, methods of PT measurement, diagnostic methods for malaria, and *Plasmodium* species, which could affect the comparison of PT among different studies. Moreover, most of the included studies used cross-sectional designs, limiting the ability to establish causality between malaria infection and increased PT. Finally, the limited number of studies and their geographic concentration also pose challenges in generalizing the findings globally.

For future research implications, it is recommended to perform more robust studies, such as randomized controlled trials or prospective cohort studies, which can help to provide a more accurate assessment of the PT in malaria patients and reduce bias. It would also be beneficial to include a more diverse sample, both in terms of geographical distribution and characteristics of patients, to allow for a broader understanding of the relationship between malaria and PT. There is also a need to investigate the exact biological mechanisms driving the observed differences in PT in patients with malaria, as this could provide a more in-depth understanding of the disease's pathophysiology and possibly lead to the development of novel therapeutic interventions.

## Conclusion

The systematic review and meta-analysis revealed PT prolongation in malaria infections caused by *P. falciparum* and *P. vivax*, particularly in severe cases, yet found no significant difference in PT between the two species. The ambiguous results concerning PT in fatal versus non-fatal cases highlight existing research gaps. Future studies should focus on refining PT measurement methods, exploring the impact of treatment and comorbidities on PT, and unraveling the molecular mechanisms of coagulation alterations induced by malaria. Advancements in these areas promise to inform targeted therapies for malaria-associated coagulopathy, aiming to improve patient outcomes.

### Supplementary Information

Supplementary Information 1.

Supplementary Information 2.

Supplementary Information 3.

Supplementary Information 4.

Supplementary Information 5.

Supplementary Information 6.

Supplementary Information 7.

Supplementary Information 8.

Supplementary Information 9.

## Data Availability

All data relating to the present study are available in this manuscript and supplementary files.
