# Supplementary material for: Alteration of prothrombin time in Plasmodium falciparum and Plasmodium vivax infections with different levels of severity: a systematic review and meta-analysis
Source: Sci Rep. 2024 May 2;14:9816. doi: 10.1038/s41598-024-60170-y (PMC11066112; doi:10.1038/s41598-024-60170-y)
Supplement: Supplementary file 3 — Supplementary Information 3. [file 41598_2024_60170_MOESM3_ESM.pdf]

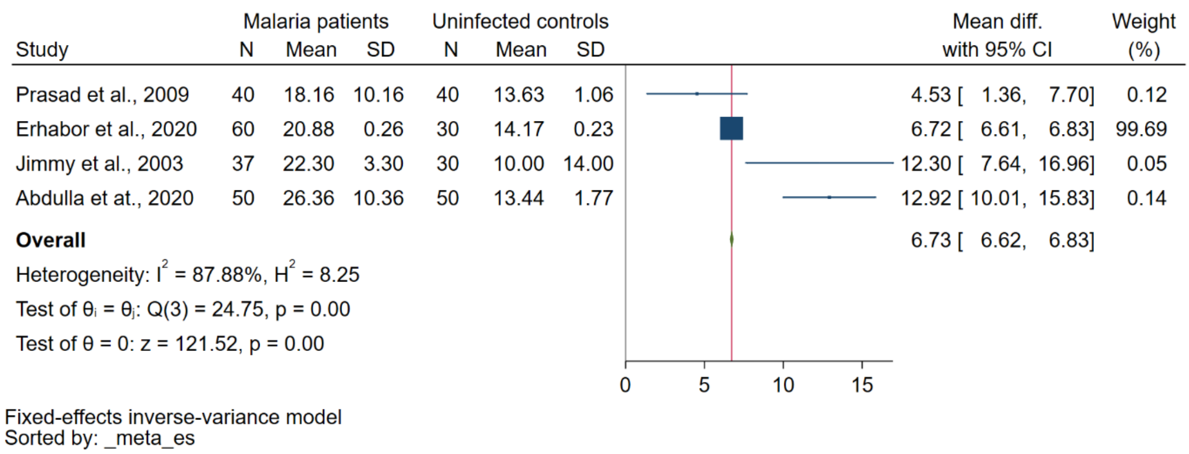

Supplementary Figure 3. The forest plot showing the difference in PT between malaria patients and uninfected individuals by the fixed-effect model. Abbreviation: CI, confidence interval; Mean Diff., mean difference; N, number of participants; SD, standard deviation.
