# Supplementary material for: Alteration of prothrombin time in Plasmodium falciparum and Plasmodium vivax infections with different levels of severity: a systematic review and meta-analysis
Source: Sci Rep. 2024 May 2;14:9816. doi: 10.1038/s41598-024-60170-y (PMC11066112; doi:10.1038/s41598-024-60170-y)
Supplement: Supplementary file 4 — Supplementary Information 4. [file 41598_2024_60170_MOESM4_ESM.pdf]

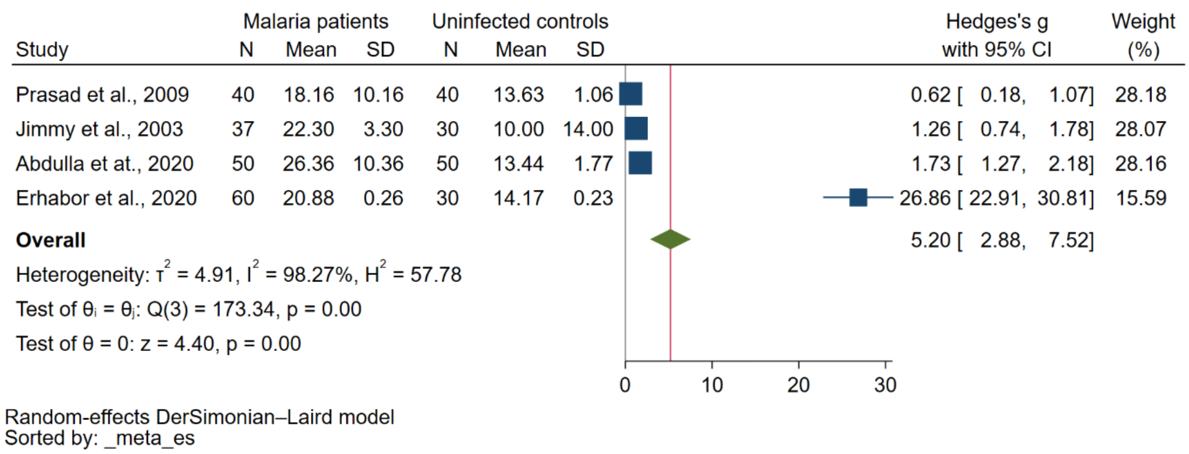

Supplementary Figure 4. The forest plot showing the difference in PT between malaria patients and uninfected individuals using SMD as the effect estimate. Abbreviation: CI, confidence interval; N, number of participants; SD, standard deviation.
