# Supplementary material for: Alteration of prothrombin time in Plasmodium falciparum and Plasmodium vivax infections with different levels of severity: a systematic review and meta-analysis
Source: Sci Rep. 2024 May 2;14:9816. doi: 10.1038/s41598-024-60170-y (PMC11066112; doi:10.1038/s41598-024-60170-y)
Supplement: Supplementary file 6 — Supplementary Information 6. [file 41598_2024_60170_MOESM6_ESM.docx]

**Table S1. Search term.** Search terns and final date of searches in different databases.

| **Databases** | **Search terms/Search strategy** | **Date (final date of searches)** |
| --- | --- | --- |
| PubMed | (coagulation OR "blood clotting" OR “prothrombin time” OR prothrombin OR “Russell viper venom time” OR “Russells viper venom time” OR “thrombotest” OR “quick test”) AND (Plasmodium OR “Remittent Fever” OR “Marsh Fever” OR Paludism)  Search results: 833 | 2 March 2023 |
| Scopus | (TITLE-ABS-KEY (coagulation OR "blood clotting" OR "prothrombin time" OR prothrombin OR "russell viper venom time" OR "russells viper venom time" OR "thrombotest" OR "quick test" ) ) AND ( TITLE-ABS-KEY ( plasmodium OR "remittent fever" OR "marsh fever" OR paludism ))  Search results: 489 | 2 March 2023 |
| EMBASE | ('coagulation'/exp OR coagulation OR 'blood clotting'/exp OR 'blood clotting' OR 'prothrombin time' OR 'prothrombin'/exp OR prothrombin OR 'russell viper venom time'/exp OR 'russell viper venom time' OR 'russells viper venom time' OR 'thrombotest'/exp OR 'thrombotest' OR 'quick test'/exp OR 'quick test') AND (plasmodium OR 'remittent fever' OR 'marsh fever' OR paludism)  Search results: 665 | 2 March 2023 |
| Ovid | (coagulation OR "blood clotting" OR “prothrombin time” OR prothrombin OR “Russell viper venom time” OR “Russells viper venom time” OR “thrombotest” OR “quick test”) AND (Plasmodium OR “Remittent Fever” OR “Marsh Fever” OR Paludism) {Including Limited Related Terms}  Search results: 465 | 2 March 2023 |
| Medline | (coagulation OR "blood clotting" OR “prothrombin time” OR prothrombin OR “Russell viper venom time” OR “Russells viper venom time” OR “thrombotest” OR “quick test”) AND (Plasmodium OR “Remittent Fever” OR “Marsh Fever” OR Paludism)  Search results: 315 | 2 March 2023 |

Note. The “/exp” indicates that it's an “explosion” in Emtree.
