# Supplementary material for: Alteration of prothrombin time in Plasmodium falciparum and Plasmodium vivax infections with different levels of severity: a systematic review and meta-analysis
Source: Sci Rep. 2024 May 2;14:9816. doi: 10.1038/s41598-024-60170-y (PMC11066112; doi:10.1038/s41598-024-60170-y)
Supplement: Supplementary file 9 — Supplementary Information 9. [file 41598_2024_60170_MOESM9_ESM.docx]

**Table S4. Meta-regression results**

| **Meta-analysis of PT** | **Covariates** | ***P* value** | **tau2** | **I^2^ (%)** | **R-squared (%)** | **Number of studies** |
| --- | --- | --- | --- | --- | --- | --- |
| **Severe vs. non-severe malaria** | Publication years | 0.501 | 4.328 | 97.78 | 0.00 | 7 |
|  | Study design | N/A | N/A | N/A | N/A | 7 |
|  | Country | 0.501 | 4.328 | 97.78 | 0.00 | 7 |
|  | Continent | 0.253 | 3.613 | 97.50 | 2.44 | 7 |
|  | Age group | 0.821 | 4.728 | 98.42 | 0.00 | 7 |
|  | *Plasmodium* species | N/A | N/A | N/A | N/A | 7 |
|  | Diagnostic method for malaria | 0.215 | 3.29 | 97.47 | 11.15 | 7 |
|  | Method for PT measurement | 0.302 | 4.482 | 98.47 | 0.00 | 7 |

N/A: not assessed because of collinearity
